# Healing of Open Upper Lip Vermillion Wounds Irradiated with CO_2_ Laser Immediately After Injury

**DOI:** 10.1089/photob.2020.4912

**Published:** 2021-09-21

**Authors:** Hiroshi Fukuoka, Nobuko Fukuoka, Yuki Daigo, Erina Daigo, Masatsugu Ishikawa, Toshiro Kibe

**Affiliations:** ^1^ Fukuoka Dental Office, Kagoshima, Japan.; ^2^ Department of Geriatric Dentistry, Osaka Dental University, Osaka, Japan.; ^3^ Department of Anesthesiology, Osaka Dental University, Osaka, Japan.; ^4^ Bees Dental Office, Fukuoka, Japan.; ^5^ Department of Oral and Maxillofacial Surgery, Field of Oral and Maxillofacial Surgery, Developmental Therapeutics Course, Graduate School of Medical and Dental Sciences, Kagoshima University, Kagoshima, Japan.

**Keywords:** lip vermillion, open wound, wound healing, pain control, CO_2_ laser

## Abstract

***Objective:*** To evaluate the healing of open upper lip vermillion wounds irradiated with CO_2_ laser immediately after injury.

***Background:*** There are reports of using CO_2_ laser therapy on healed wounds for scar tissue reduction. However, limited data exist regarding its use immediately after an injury. Thus, the role of CO_2_ laser in wound healing remains unclear.

***Methods:*** Two patients with open upper lip vermillion wounds were treated using CO_2_ laser irradiation to the area postsuturing.

***Results:*** Good functional and aesthetic results were obtained from the therapy, with no postoperative pain complaints or infection.

***Conclusions:*** CO_2_ laser irradiation, performed immediately after an injury, could be an effective treatment option for open vermillion wounds.

## Introduction

The lips are the part of the face that is most vulnerable to injury, and although conservative treatment is possible if the tissue defect is small, large defects require surgery.^1,2^ Recently, treatment with wound dressings has enabled epithelial regeneration by covering the epithelial defect and maintaining moisture with blood clot or exudate to promote the formation of granulation tissue.^3,4^ There are various wound dressings available, with researchers investigating methods that use biomaterials and growth factors.^3^ These therapies not only alleviate pain and prevent wound infections, but they also promote early wound closure and suppress scar formation.^3,4^ However, even if a wound dressing is used, the granulation tissue generated during the healing process may become fibrotic, resulting in the formation of scar tissue and scar contracture.^4^

Recently, lasers have been used in the surgical treatment of oral tissues^5,6^ and photobiomodulation (PBMT) has been applied to promote wound healing.^7^ CO_2_ lasers have been used after wound healing to shrink scar tissue. It acts on the granulation tissue that forms in the early stages after surgery.^8,9^ As an advantage of utilizing irradiating CO_2_ laser at an early stage, it is possible to treat while coagulating blood so that the operative field is clear,^5,6^ and the wound can be dressed.^5,6^ Therefore, the risk of infection and pain is low.^5,6^ Further, it can be applied to children^6^ and can minimize scar tissue formation.^10,11^

However, we could find no clinical report of using a CO_2_ laser immediately after an injury. In this study, we report two cases in whom CO_2_ laser irradiation was performed immediately after an injury to the upper lip vermillion with favorable results.

## Materials and Methods

### Case 1

**FIG. 1.**
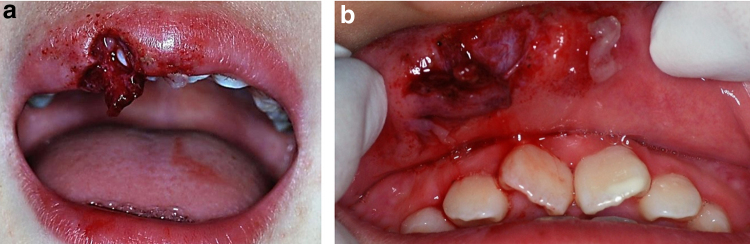
Extraoral and intraoral findings in case 1 after injury to the upper lip. **(a)** Outer upper lip, **(b)** inner upper lip.

**FIG. 2.**
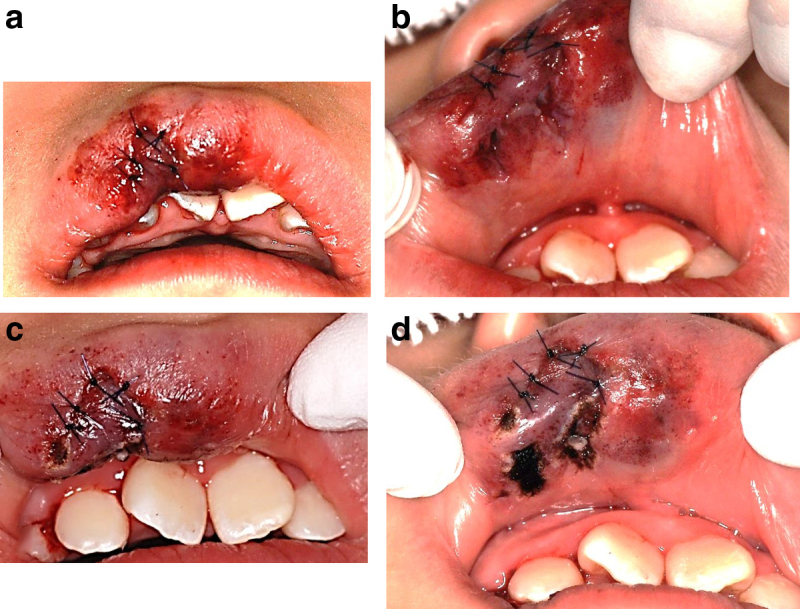
Treatment for upper lip laceration injury in case 1. **(a)** Outer upper lip sutures, **(b)** inner upper lip sutures. **(c)** After laser irradiation of the outer upper lip. **(d)** After laser irradiation of the inner upper lip.

A 9-year-old boy presented to our hospital 30 min after a playground injury to the upper lip. On examination, a contaminated 20 mm long laceration with soft tissue defect was observed on the right side of the upper lip vermillion (Fig. 1a, b). It was associated with severe pain on touching and mild bleeding. The patients' verbal informed consent for treatment was recorded on the medical chart. After infiltrating anesthesia around the wound, minimal debridement was performed and the wound was sutured with 5-0 nylon suture (Fig. 2a, b). To manage the dead space caused due to extensive tissue loss, blood was allowed to accumulate in the area. A carbonized layer was created with the application of high-intensity laser therapy (HILT) on the blood (Fig. 2c, d). To prevent infection, the wound surface was treated with an antibacterial (Sawacillin 250 mg, 3 caps after each meal × 4 days) and an anti-inflammatory analgesic (Calonal 200 mg, 6T at 2T per dose × 3 days). Details regarding the laser equipment and the irradiation parameters are listed in Table 1. The patient was instructed to avoid wiping the wound and to restrict lip movement. PBMT was undertaken at follow- up visits after disinfection with Germitol and iodine to promote healing. Parameters for the same are enlisted in Table 1.

**Table 1.** Details of Parameters Used in Case 1

Parameter	HILT	PBMT
Power density (W/cm^2^)	356	19.1
Energy density (J/cm^2^)	64,080	3438
Duration of treatment (sec)	180	180
Frequency of treatment	At the first visit	1, 5, 12, 19, and 30 days after surgery
Cumulative dose (J)	498	27

HILT, high-intensity laser therapy; PBMT, photobiomodulation.

### Case 2

**FIG. 3.**
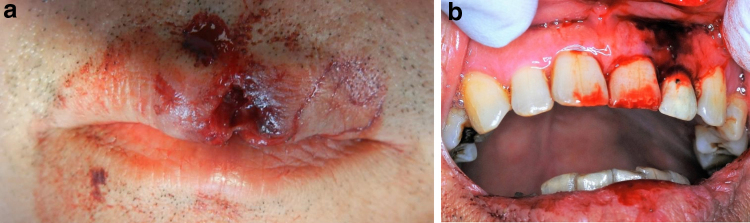
Extraoral and intraoral findings in case 2 after injury to the upper lip. A laceration was observed piercing through the upper labial gingiva. **(a)** Outer upper lip, **(b)** inner upper lip.

**FIG. 4.**
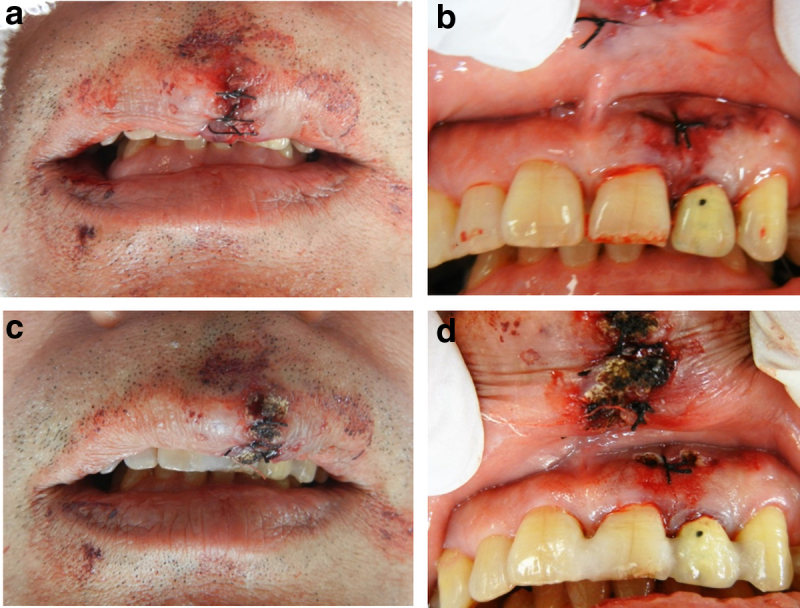
Treatment for upper lip laceration injury in case 2. **(a)** Sutures on the outer upper lip. **(b)** Sutures on the inner upper lip and gingiva. **(c)** Laser irradiation of the outer upper lip. **(d)** Laser irradiation of the inner upper lip and gingiva.

A 66-year-old male patient was brought to the emergency room with injuries on the upper lip and labial gingiva, which occurred while cutting bamboo. The offending piece of bamboo was removed. Preliminary examination revealed no other signs of injury. The patient was then referred to our hospital where he arrived 2 h after the injury. On examination, a 3 cm long laceration with soft tissue defect was observed on the left upper lip (Fig. 3a). The wound penetrated to the gingiva in relation to the upper left incisors (Fig. 3b). Initial steps of management were repeated as in case 1. An additional suture was required on the gingival wound (Fig. 4). HILT was undertaken with the parameters listed in Table 2. Antibiotic, as before, and analgesic (Loxonin 60 mg, 3T × 3 days) were prescribed. A gargle (4% Azunol gargle, 10 mL diluted in water; after each meal) was added. Postoperative instructions were given as in case 1. PBMT was undertaken at follow-up visits and parameters are listed in Table 2.

**Table 2.** Details of Parameters Used in Case 2

Parameter	HILT	PBMT
Power density (W/cm^2^)	356	19.1
Energy density (J/cm^2^)	106,800	3438
Duration of treatment (sec)	300	180
Frequency of treatment	At the first visit	1, 7, 14, and 30 days after surgery
Cumulative dose (J)	830	27

HILT, high intensity laser therapy; PBMT, photobiomodulation.

### Laser equipment and details of parameters

In this study, 10.6 μm CO_2_ laser (Takara Belmont CO., Ltd., Japan) was used with a short ceramic tip (ϕ: 1.0 mm). For HILT, we referred to the manufacturer's recommended value that allowed for efficient blood coagulation while taking care not to cause tissue damage due to over irradiation. HILT was performed for ∼3 min in case 1 and for ∼5 min in case 2 using a power of 3 W irradiation mode: on time, 0.060 sec; off time, 0.005 sec in super pulse mode. The distance from the wound was 2–3 mm. Therefore, the total energy delivered in case 1 was 498 J and that in case 2 was 830 J.

PBMT was performed at subsequent visits with the goal of promoting healing.^7,10,11^ Irradiation was performed at 3 W for 3 min using the BP mode at a distance of ∼2–3 mm from the wound surface, such that the total energy delivered was ∼27 J). The BP mode is a mode created by the manufacturer exclusively for PBMT, and the pulse width and pulse duration are specified values (on: 0.001 sec, off: 0.019 sec). Since the pulse width is very short, no thermal effect occurs.

## Results

### Case 1

**FIG. 5.**
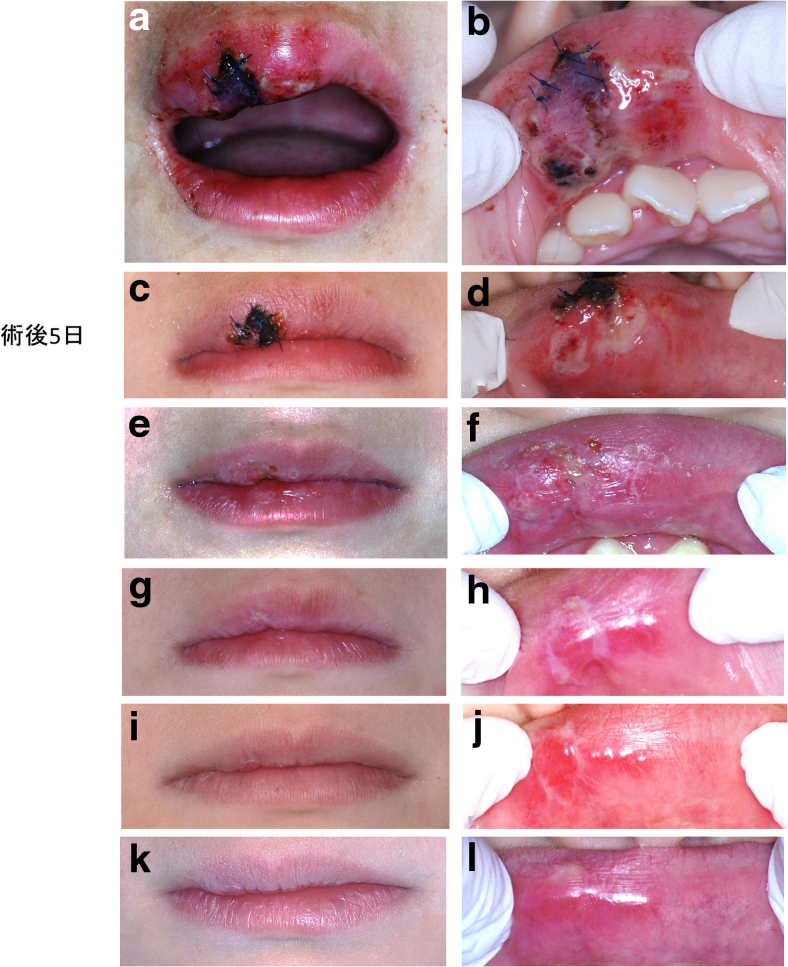
Case 1 healing process **(**outer upper lip; **a, c, e, g, i, k,** inner upper lip; **b, d, f, h, j, l)**. **(a, b)** One day after surgery, the outer surface of the wound is covered by a carbonized layer, and the inner wound surface is covered by a fibrin layer. Swelling of the injured upper lip is evident. **(c, d)** Five days after surgery, the clots and carbonized layer of the outer wound surface are replaced by a scab. **(e, f)** Twelve days after surgery, removing the scab exposed slight depressions of the upper lip wound surface. **(g, h)** Nineteen days after surgery, the depression of the upper lip wound surface has shrunk compared with 1 week earlier. **(i, j)** One month after surgery, the outer part has almost completely healed. However, a small amount of scar tissue is still present on the inside. **(k, l)** Two months after surgery, the inner scar tissue has shrunk.

Swelling was observed at the first follow-up, 1 day after the treatment (Fig. 5a). The outer surface of the wound was covered by a carbonized layer, and the inner surface by a fibrin layer (Fig. 5b).

At the second follow-up, 5 days after the treatment, the swelling was found to have decreased, the carbonized layer had ben replaced by a scab (Fig. 5c) and the fibrin layer had shrunk (Fig. 5d). Owing to an apparent scar, tranilast (one capsule of 100 mg Rizaben twice daily after breakfast and dinner) was prescribed for 2 weeks.^8^

Suture removal was undertaken 1 week later to reveal slight depressions (Fig. 5e, f), which became smaller at the next follow-up (Fig. 5g, h). Ten days later, almost complete healing was observed although scarring was still apparent (Fig. 5i, j).

The scar tissue had shrunk and no aesthetic, sensory, or motor abnormality could be detected 2 months after the treatment was initiated (Fig. 5k, l).

### Case 2

**FIG. 6.**
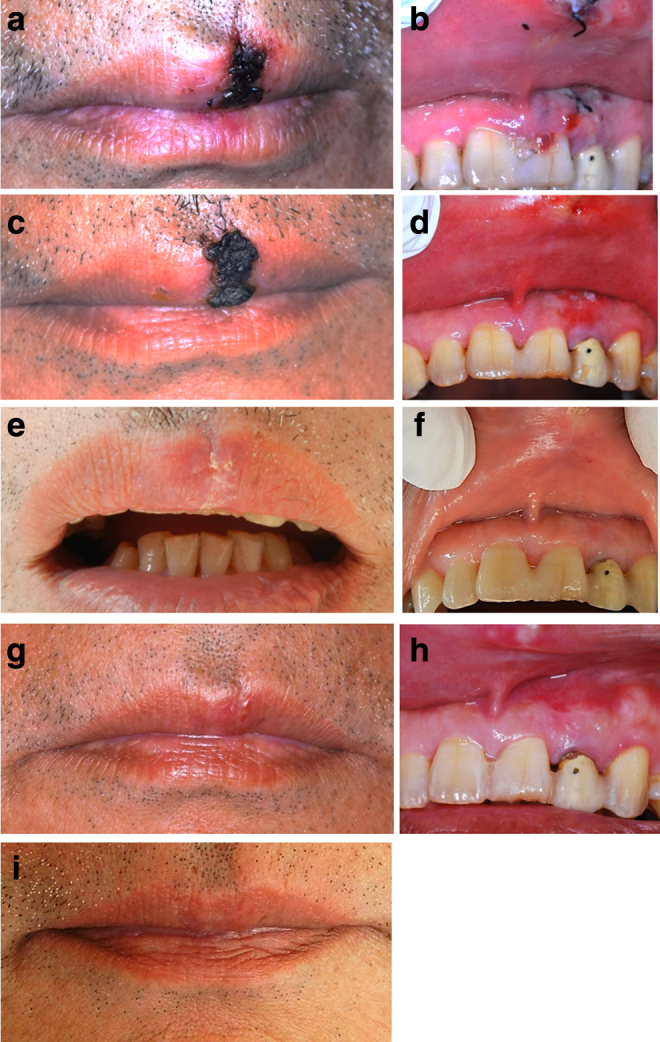
Case 2 healing process **(**outer upper lip; **a, c, e, g, i,** inner upper lip; **b, d, f, h)**. **(a, b)** One day after surgery, the outer wound surface is covered with a carbonized layer and the inner wound with a fibrin layer. No redness or swelling seen. **(c, d)** One week after surgery, the clots and carbonized layer on the external wound surface have been replaced by a scab. The inner wound surface is covered with granulation tissue and almost closed. **(e, f)** Two weeks after surgery, the scab on the upper lip has fallen off spontaneously and the gingiva is covered with healthy mucosa. **(g, h)** One month after surgery, the upper lip and gingiva are almost completely healed. However, a mass that indicated scar tissue formation was palpable deep in the upper lip. **(i)** Six months after surgery, the mass deep in the upper lip has disappeared and the upper lip injury had no aesthetic problems.

Similar observations were made as in case 1; however, there was no redness, scarring, or reported pain (Fig. 6a, b).

One week after the treatment, the outer surface was found to be carbonized and replaced with a scab (Fig. 6c). The gingival wound had almost completely closed (Fig. 6d). Suture removal was performed.

Two weeks after surgery, the patient reported no sensory or motor disturbance. The scab had fallen off spontaneously (Fig. 6e). In addition, the gingiva was covered with healthy mucosa and there was hardly any scarring (Fig. 6f).

One month after surgery, the injured lip and the gingiva were almost completely healed with no functional or aesthetic problem. However, a mass that indicated scar tissue formation was palpable deep (Fig. 6g, h); hence, tranilast was prescribed.^8^

Six months after surgery, tranilast was discontinued because there was no evidence of scarring (Fig. 6i).

## Discussion

In this study, we described our experience with two cases in which CO_2_ laser irradiation was used on open upper lip vermillion wounds immediately after injury. Healing was uneventful with no pain or infection.

For tissue defects in the vermillion, where the width of the defect is less than half the width of the lip, a procedure called vermillion advancement flap is used, which involves creating a skin flap containing the labial artery from the adjacent vermillion and then advancing it to repair the defect.^2^ Conversely, a procedure called V-Y advancement is used for upper lip defects covering more than half the lip width.^2^ However, postoperative pain and infections are unavoidable with both procedures.

Recently, surgical treatment options with lasers have been reported to provide healing without infection, pain, or other discomfort, and minimize the formation of scar tissue.^5,6^ Therefore, we used a CO_2_ laser on wounds with tissue defects to form a protective film with a blood coagulation layer by creating a carbonized layer. This replicates a wound tissue covering and could be an effective form of dressing therapy.^3,4^ Suturing was performed with the hope of achieving primary healing in the area where the wound edges matched and there was no tissue defect. As a result, these patients experienced almost no subjective symptoms, and their healing courses were good and free of infection. Moreover, there were no obvious aesthetic or functional problems on the surface of the lip. These results resembled those of a clinical report^5,6^ in which there was almost no pain, infection, or scar tissue formation after periodontal surgery with a CO_2_ laser, and healing was achieved without the use of packs or other protective films. Further, Fukuoka et al.^10^ and Daigo et al.^11^ reported that on healing of the mucosal epithelium by CO_2_ laser irradiation of blood on the wound surface after tooth extraction created a protective film with a blood coagulation layer, which significantly reduced the expression of myofibroblasts that contribute to scar tissue formation. This method of laser irradiation of open wounds such as after tooth extraction resembles the procedure we used in the present cases. When a laser diode was used to irradiate the extracted site, the extracted tooth socket had less expression of the myofibroblasts (expressing α-smooth muscle actin) involved in scar formation at both the surface layer and deep layer.^11^ In contrast, the CO_2_ laser had fewer expressing α-SMA surface layers of the tooth extraction wound than the control group.^10,11^ This is the relationship between tissue permeability and laser wavelength. In this case report, we decided to use Rizaben (tranilast) along with the CO_2_ laser to prevent scarring in the deep layer.

From the mentioned, because the depth of CO_2_ laser is shallow, its effects may not have reached the deeper tissues. Hence, we also used drug therapy. In case 2, it is possible that the appearance of the scar tissue was effectively suppressed by the early administration of tranilast.

## Conclusions

Using HILT with a CO_2_ laser on open wounds with tissue defects carbonizes the surface of the blood accumulated in the tissue defect. This carbonized layer creates a protective film with a blood coagulation layer that can suppress pain and reduce wound infections.
